# Adoption, implementation, and sustainability of early childhood feeding, nutrition and active play interventions in real-world settings: a systematic review

**DOI:** 10.1186/s12966-023-01433-1

**Published:** 2023-03-20

**Authors:** Rivka Gelman, Jillian Whelan, Sheree Spiteri, Danijela Duric, Winnie Oakhill, Samuel Cassar, Penelope Love

**Affiliations:** 1 School of Exercise and Nutrition Science, Deakin University, Geelong, VIC 3220 Australia; 2 School of Medicine, Institute of Health Transformation, Deakin University, Geelong, VIC 3220 Australia; 3 Institute for Physical Activity and Nutrition, School of Exercise and Nutrition Science, Deakin University, Geelong, VIC 3220 Australia; 4 Centre for Youth Mental Health, University of Melbourne, Melbourne, VIC 3052 Australia

**Keywords:** Implementation science, Intervention adoption, implementation and sustainability, Early childhood nutrition and active play

## Abstract

**Background:**

Instilling healthy dietary habits and active play in early childhood is an important public health focus. Interventions supporting the establishment of nutrition and active play behaviours in the first years of life have shown positive outcomes and long-term cost-effectiveness, however, most are research trials, with limited evidence regarding real-world application. Implementation science theories, models and frameworks (TMFs) can guide the process of research translation from trial to real-world intervention. The application of TMFs within nutrition and active play intervention studies in early childhood (< 5 years) is currently unknown. This systematic review identified the use of TMFs and barriers/ enablers associated with intervention adoption, implementation, and sustainability in early childhood nutrition and active play interventions implemented under real-world conditions.

**Methods:**

Six databases were searched for peer-reviewed publications between 2000–2021. Studies were included if primary outcomes reported improvement in diet, physical activity or sedentary behaviours amongst children aged < 5 years and interventions were delivered under real-world conditions within a community and/or healthcare setting. Two reviewers extracted and evaluated studies, cross checked by a third and verified by all authors. Quality assessment of included studies was completed by two authors using the Mixed Methods Appraisal Tool (MMAT).

**Results:**

Eleven studies comprising eleven unique interventions were included. Studies represented low, middle and high-income countries, and were conducted across a range of settings. Five TMFs were identified representing four of Nilsen’s implementation model categories, predominantly ‘evaluation models’. Ninety-nine barriers/facilitators were extracted across the three intervention phases—Implementation (*n* = 33 barriers; 33 facilitators), Sustainability (*n* = 19 barriers; *n* = 9 facilitators), Adoption (*n* = 2 barriers; *n* = 3 facilitators). Identified barriers/facilitators were mapped to the five domains of the Durlak and DuPre framework, with ‘funding’, ‘compatibility’ and ‘integration of new programming’ common across the three intervention phases.

**Conclusions:**

Findings demonstrate that there is no systematic application of TMFs in the planning, implementation and/or evaluation of early childhood nutrition and active play interventions in real-world settings, and selective and sporadic application of TMFs occurs across the intervention lifespan. This apparent limited uptake of TMFs is a missed opportunity to enhance real-world implementation success.

**Trial registration:**

PROSPERO (CRD42021243841).

**Supplementary Information:**

The online version contains supplementary material available at 10.1186/s12966-023-01433-1.

## Contributions to the literature

• This systematic review is the first to identify the use of implementation science theoretical approaches [theories, models and frameworks (TMFs)] in early childhood (< 5 years of age) nutrition and physical activity public health interventions.

• Findings demonstrate limited systematic application of existing TMFs and highlight the challenge of author-created single use tools, which create additional tools without necessarily strengthening the quality or validity of existing TMFs.

• The extensive identification of facilitators and barriers across the intervention lifespan provides opportunities to enhance success of early childhood interventions under real-world conditions, especially regarding funding, compatibility and integration into routine practice.

## Background

Early childhood provides a critical window of opportunity to establish heathy food preferences and dietary habits [1]. The World Health Organisation (WHO) emphasizes the importance of optimal nutrition in early life to foster healthy growth and development [2]. Optimal nutrition in early childhood suggests a diet high in whole grains, fruit and vegetables, with limited intakes of foods/drinks that contain excess sodium, fat, and sugar, to promote lifelong healthy eating [3]. Instilling healthy dietary habits in early childhood is therefore an important public health focus. Recent changes in population dietary patterns, including increased consumption of ultra-processed foods, may promote unhealthy eating behaviours and preferences that track into adolescence and adulthood [4] which have been associated with all-cause mortality [5]. A synthesis of several global data sources reveals suboptimal feeding practices among young children, with only 42% of children < 6 months being exclusively breastfed, and 20% of children aged 6–23 months consuming diets with low diversity and frequency [6]. Children under age 5 years were found to consume diets low in nutritious foods such as fruits, vegetables, animal foods and fortified foods [6].

Advocating for healthy nutrition and physical activity in children at a young age is essential to establish a strong foundation for a long-term healthy lifestyle. Research has shown that although feeding and physical activity behaviours that have been developed in early childhood can be modified [7], it is imperative to encourage healthy behaviours as early as possible before they become ingrained [8, 9]. Once a child matures into adulthood, behaviour changes required to shift to a healthier lifestyle are more challenging [10]. Interventions supporting the establishment of nutrition and healthy eating behaviours in the first years of life have been found to be cost-effective [11], however, few interventions specifically target early childhood, with most targeting children aged 6–17 years [12].

Children are also exposed to a range of environments during their childhood which play a significant role in influencing their health and development [2], including the home, family and other caregivers, educational services including childcare and school, and the community setting. These environments provide different dimensions of context, such as organisational support, financial resources and physical infrastructure, all of which can impact implementation outcomes [13]. Context evolves over time and should therefore be considered across the life of an intervention, including the development of intervention components, implementation and sustainability strategies, and scale-up [14]. There is limited guidance however for real-world implementation of setting-specific initiatives with different contexts, for example childcare in a rural area compared with an urban area [15]. Furthermore, many interventions developed in recent years have been implemented as research trials, with limited evidence available to provide insights into real-world application outside of controlled research settings [16–19].

To foster the uptake of interventions under real-world conditions, it is imperative to gain insight into how they can be delivered outside of controlled research conditions and effectively scaled up [20]. The field of implementation science and its use of ‘theoretical approaches’ (referred to as theories, models and frameworks, TMFs) [21] can guide this process of research translation from intervention design through to sustained practical real-world application [22]. An implementation theory assists with the overarching planning and evaluation activities; an implementation model provides conceptual guidance to researchers and practitioners through multiple stages; and an implementation framework provides organizational structure to the work [23]. There are also a myriad of barriers and facilitators affecting successful implementation of interventions in real-world conditions, and as such, it is important to examine these factors at each stage of adoption, implementation, and sustainability [22, 24, 25], where adoption refers to the uptake of an intervention by an organisation, implementation refers to the delivery of an intervention by an organisation [25], and sustainability refers to the enduring administration of an intervention and ability to successfully integrate it into usual practice within an organisation [26].

Whilst there is a high degree of awareness surrounding the usefulness of various implementation TMFs, the prevalence of employing such approaches in intervention studies on early childhood feeding, nutrition and active play is currently not known [22, 24]. Therefore, this systematic review aims to identify (a) the use of implementation science theories, models and/or frameworks (TMFs) and (b) barriers and facilitators associated with the adoption, implementation, and sustainability; in early childhood (< 5 years) feeding, nutrition and active play interventions implemented under real-world conditions. Findings will provide insights into the selection of TMFs and their application across the lifespan of early childhood intervention studies, to guide the transfer of research into real-world settings and contexts.

## Methods

This systematic review was prospectively registered with PROSPERO (CRD42021243841) and undertaken in accordance with the Preferred Reporting Items for Systematic Reviews and Meta-Analyses (PRISMA) guidelines [27] (Additional file 2).

### Data sources, search terms and eligibility criteria

Six online databases MEDLINE, EMBASE, CINAHL, PsycINFO, CENTRAL and SCOPUS were searched for English language articles published on or after 1 January 2000 to 1 July 2021.

The PICO framework was used to develop key search terms for each database search as displayed in Table 1 (see Additional file 1). As this review explored studies delivered in real-world settings, the inclusion of a control group was not necessary, and as such, no PICO search terms were included under the comparison/control group category.

**Table 1:** Search terms developed using the PICO framework

**P**: **P**atient/ **P**opulation/ **P**roblem Parents with young children (<5years old); focusing on feeding, nutrition and active play	Infan* or “early childhood” or child* AND feed* or “feeding practice*” or diet* or nutrition or “physical activity” or “sedentary time” or active* or play or BMI or “obesity prevention” or “healthy lifestyle*” or “energy balance”
**I/E: I**ntervention/ **I**ndicator/ **E**xposure/ **E**vent Adoption, implementation, sustainability and scale up of interventions in real world context	program* or intervention or initiative AND implement* or disseminat* or diffus* or delivery or uptake or adopt* or adapt* or modif* or utilisation or utilization or sustain* or feasib* or evaluat* AND “scale up” or “scaled up” or “scaling up” or scaling or scalability or “scale out” or translat* or “roll out” or “rolled out” or “real-world” or “research to practice” or ‘routine practice’ or ‘service delivery’
**C: C**omparison/ **C**ontrol	n/a
**O: O**utcome Theories, frameworks, models used for implementation Barriers and facilitators associated with adoption, implementation and sustainability of interventions delivered in real world context	framework* or model*or plan* or approach* or strateg* or protocol* or guideline* or manual* or concept*

Studies were eligible for inclusion if: i) primary outcomes demonstrated improvement in diet, physical activity or sedentary behaviours amongst children aged < 5 years; ii) the intervention was delivered within a community and/or healthcare setting, and iii) the intervention was delivered under real-world conditions, to capture implementation, translation, dissemination, effectiveness and scale-up studies. Studies were excluded if: i) they were efficacy trials or ii) they tested or applied policies as a single component intervention.

#### Study selection

Article titles and abstracts were screened by two authors (RG, WO). Subsequent full text screening was completed by two authors (RG, SS) using the online software Covidence [28]. Any disagreement on the inclusion of a study was discussed and resolved by RG and SS, and a consensus agreement made by PL as needed.

### Data extraction and quality assessment

Extraction of study data involved two processes. Data extraction regarding study characteristics was completed by two authors (RG, DD) using Covidence [28]. Data extraction related to barriers and facilitators was completed by three authors (RG, PL, JW) using NVIVO [29]. Two excel spreadsheets were created for data analysis. Extraction of study data involved two processes. Data extraction regarding study characteristics was completed by two authors (RG, DD) using Covidence [28]. Data extraction related to barriers and facilitators was completed by three authors (RG, PL, JW) using NVIVO [29] Two excel spreadsheets were created for data analysis.

The first analysis spreadsheet recorded: study date, population, design, intervention setting and strategies, implementation TMFs, model category, model application, factors (i.e., barriers and facilitators) relating to adoption, implementation and sustainability of the implementation, and results. Implementation models were grouped in accordance to the following five categories described by Nilsen et al. [21]. These are: process models’ used to guide the implementation; ‘determinant frameworks’ used to understand outcome influences; ‘classic theories’, used to understand implementation aspects; ‘implementation theories’ used to understand implementation features; and ‘evaluation frameworks’, used to assess relevant and successful implementation features [21]. The study’s application of the implementation model was also noted, with one or a combination of any of the three following applications possible: ‘designing the intervention’, ‘planning the intervention evaluation’, and ‘interpreting the intervention results’.

The second analysis spreadsheet based on the Durlak and DuPre framework [30] was developed to identify barriers and facilitators across the three phases of an intervention; adoption, implementation and sustainability [31, 32]. Extraction of data regarding barriers and facilitators to the adoption, implementation and sustainability of each intervention was categorised according to the five domains within Durlak and DuPre’s framework [30], namely: ‘community level factors’, ‘provider characteristics’, ‘characteristics of the innovation’, ‘prevention delivery system: organisational capacity’ and ‘prevention support system’.

Quality assessment of selected studies was completed by two authors (RG, DD) using the Mixed Methods Appraisal Tool (MMAT) [33]. The MMAT was selected due to its suitability in appraising varying study designs, including qualitative, quantitative and mixed methods. As per the eligibility criteria of this review, appraisal questions relating to qualitative, quantitative non-randomised, quantitative descriptive, and mixed methods study designs were completed. Unlike other quality appraisal tools, the MMAT discourages the use of an overall score. The MMAT rates different categories against ‘Yes’, ‘No’ or ‘Can’t tell’. See Additional file 3.

## Results

### Study selection

**Fig. 1:**
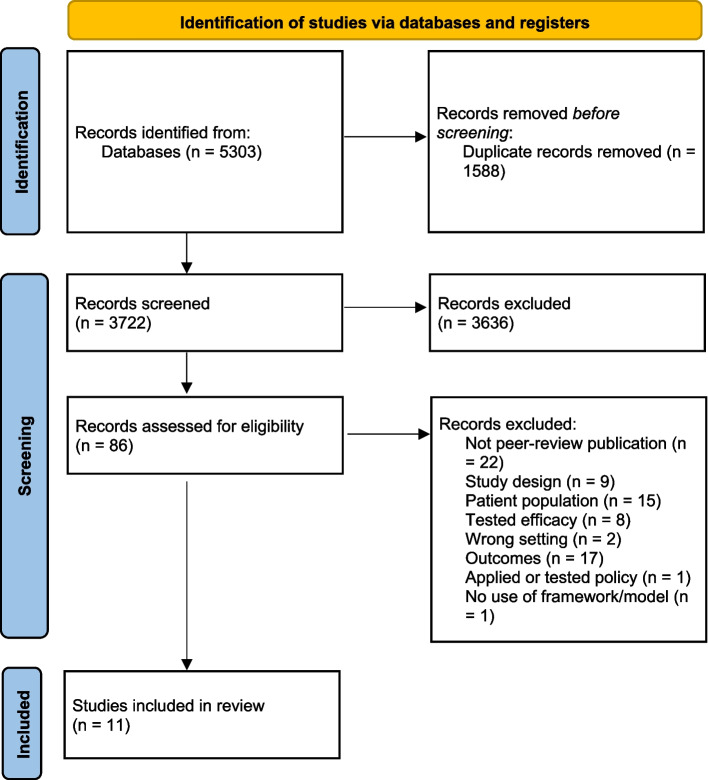
Search strategy and inclusion guided by the PRISMA Flow Diagram

The study selection is outlined in Fig. 1. A total of 5303 articles were identified from the six database searches, with 1588 articles removed as duplicates. Screening titles and abstracts of the remaining 3722 articles resulted in 3636 exclusions, leaving 86 articles eligible for full text review. Full text screening excluded 75 articles due to not being a peer-review publication; incorrect patient population, outcomes or study design; efficacy studies; studies applying or testing a policy; and studies not including the use of a framework/model. A total of eleven studies comprising eleven unique interventions, met all eligibility criteria and were included in this review [34–44].

### Study characteristics

Table 2 provides an overview of the included studies, including intervention strategies, implementation models and results. In summary, of the eleven studies included, nine were mixed methods [35–41, 43, 44] and two used a qualitative design [34, 42]. One paper was a hybrid effectiveness-implementation trial [44]. Four of the eleven studies were conducted in the United States [34, 37, 38, 44], two in Australia [40, 41], two in Bangladesh [39, 43], one in the Netherlands [36], one in Malawi [35] and one in Ethiopia [42]. The studies were conducted in a range of settings including five in community [35, 39, 41–43], four in early care and education [36, 38, 40, 44], one in a Women, Infants, and Children (WIC)-clinic in a community environment [34] and one in local parks and recreation centres [37]. No studies were conducted within a healthcare setting.

**Table 2:** Study characteristics

Author, year, country	Study Design	Study Population	Age of children in study	Intervention Setting	Implementation Model, Theory, Framework	Implementation Model Category
Sako, 2017 [41] Ethiopia	Qualitative research	52 key informant interviews, 6 senior-level stakeholder interviews, 31 focus group discussions	6-23 months	Community based, Kebele	Yamey framework	Implementation theories (in designing, planning, evaluating and interpretation of intervention results)
Norton 2021 [40] Australia	Mixed methods	26 child health nurses	2-6 years	Community health centre	The integrated Promoting Action on Research Implementation in Health Services (i-PARIHS)	Implementation theories (in designing intervention)
Eldridge 2017 [33] USA	Qualitative research	12 clinics; 47 staff	Breastfeeding children	WIC-clinics across NY State (n=12)	Created own data analysis using concepts from organisational theory and the YCDI logic model	Determinant framework and classic theories
Murtha 2021 [39] Australia	Mixed methods	80 participants from 24 early care and education and other child-related services attended the LEAPS training. 63 participants completed the evaluation	0-5 years in ECEC	Early care and education and other child-related services	Project logic model and RE-AIM	Evaluation frameworks
Moucherard 2020 [38] Bangladesh	Mixed methods	668 health workers - surveys 269 service observation checklists 218 focus groups and interviews with stakeholders and health worker supervisors -	<2 years	Household visits (Bangladesh); facility-based social franchises (Vietnam)	Developed own conceptual framework	Determinant framework And Evaluation framework
Gladstone 2018 [34] Malawi	Mixed methods	6 HSAs; 56 pre- and post-children; 20 caregiver interviews	<2 years	Community and home-based setting. Mangochi (rural) Blantyre (urban)	Medical Research Council (MRC) Framework for complex interventions	Evaluation frameworks
Luecking 2021 [37] USA	Mixed methods	Convenience sample of 92 early care and education centres (*n* = 48 intervention group, *n* = 44 delayed control group). Participants included (*n* = 189 providers, *n* = 446 parents)	3-4 years	Early care and education centres	Fidelity index	Process models
Sarma 2021 [42] Bangladesh	Mixed methods	Caregivers of children 6-59 months, community health workers, and BRAC staff members	6-59 months	164 sub-districts and 6 urban slums in Bangladesh	Program Impact Pathway (PIP)	Implementation theories (in planning intervention evaluation)
Heerman 2018 [36] USA	Mixed methods	26 parent-child (3-5 years) pairs	3-5 years	3 local parks and recreation centres	RE-AIM	Evaluation frameworks
Harms 2021 [35] Netherlands	Mixed methods	427 children (2-4 years) from 12 intervention pre-schools. In-depth parent interviews = 15, In-depth implementers interviews = 3, Questionnaire: physical home environment = 41, Questionnaire: intervention appreciatio*n* = 19	2-4 years	SuperFIT Family component: home environment. Overarching program also has a preschool component	RE-AIM	Evaluation frameworks
Swindle 2021 [43] USA	Hybrid type III Implementation study	Educators across 9 early care and education sites included in a federally funded Head Start program. 4 sites, 20 classrooms, 39 educators, and 305 children received Enhanced support. 5 sites, 18 classrooms, 36 educators, and 316 children received Basic support	<5 years	Early care and education centres (one agency)	RE-AIM	Evaluation frameworks

Quality assessment of the studies was conducted using the MMAT with scores reported in Supplementary file 1. All eleven studies included clear research questions and data to address the research questions and therefore passed the initial screening stage indicating their appropriateness. Each study [qualitative (*n* = 2) and mixed methods (*n* = 9)] was then scored against their relevant study design category. The two qualitative studies scored a ‘yes’ to all seven items [34, 42]. Comparatively, studies assessed as mixed methods were rated as lower quality with only two scoring ‘yes’ to all 17 items [39, 40].

### Application of implementation theoretical approaches—theories, models and frameworks (TMFs)

Five TMFs were identified namely, the Program Impact Pathway (PIP) [43], Medical Research Council (MRC) Framework, Yamey Framework, and The integrated Promoting Action on Research Implementation in Health Services (i-PARIHS) [41]. The most commonly identified TMF was RE-AIM [45] (Reach, Effectiveness, Adoption, Implementation, Maintenance) used across four different studies [36, 37, 40, 44]. Two studies used models created by the study authors [34, 39] and one study used a fidelity index [38]. Regarding setting and context specific use of TFMs, no pattern was observed in our review. For example, RE-AIM was used across education, recreation centres and home environments in different rural and urban contexts.

These identified TMFs represented four of Nilsen’s five implementation model categories [21]. ‘Evaluation models’ were represented most frequently (*n* = 5) [35–37, 40, 44], followed by ‘implementation theories’ (*n* = 3) [41, 43, 46], ‘determinant frameworks’ (*n* = 2) [34, 39], and ‘process models’ (*n* = 1) [38]. The three studies using ‘implementation theories’ applied these to planning the intervention evaluation (*n* = 1) [43], designing the intervention (*n* = 1) [41], and assessing the planning, design, and evaluation of the intervention (*n* = 1) [46].

### Barriers and facilitators pertinent to intervention adoption, implementation and sustainability

Of the eleven studies, three described factors relating to adoption [36, 37, 40], nine described implementation factors [34, 35, 37, 38, 40, 41, 43, 44, 46] and three described sustainability factors [37, 39, 46]. One study described factors relevant to all three phases [37], and two studies described factors relevant to two phases—adoption and implementation [40]; implementation and sustainability [46]. A total of 99 factors were identified across the three intervention phases. Factors mainly related to Implementation (*n* = 33 barriers; 33 facilitators), followed by Sustainability (*n* = 19 barriers; *n* = 9 facilitators) then Adoption (*n* = 2 barriers; *n* = 3 facilitators) (See Additional file 4).

Identified barriers and facilitators were categorised by intervention phase using the five domains of the Durlak and DuPre framework [30] (Table 3). These are: community level factors, provider characteristics, characteristics of the innovation, prevention delivery system, and prevention support system. Within these five domains, nineteen of the twenty-four factors were identified across all three intervention phases: three were identified within the adoption phase; seventeen within the implementation phase; and thirteen within the evaluation phase. Five factors were not identified across any of the three intervention phases—prevention theory and research; positive work climate; organisational norms regarding change; formulation of tasks; and staffing resources. Only three factors were identified across all three intervention phases, namely ‘funding’, ‘compatibility’ and ‘integration of new programming’. There were multiple occasions where factors were considered a barrier (due to a lack of) and a facilitator (when present), such as ‘funding’, ‘policy’, ‘perceived benefits’, ‘self-efficacy’, compatibility’, ‘communication’, and ‘leadership’.

**Table 3:**
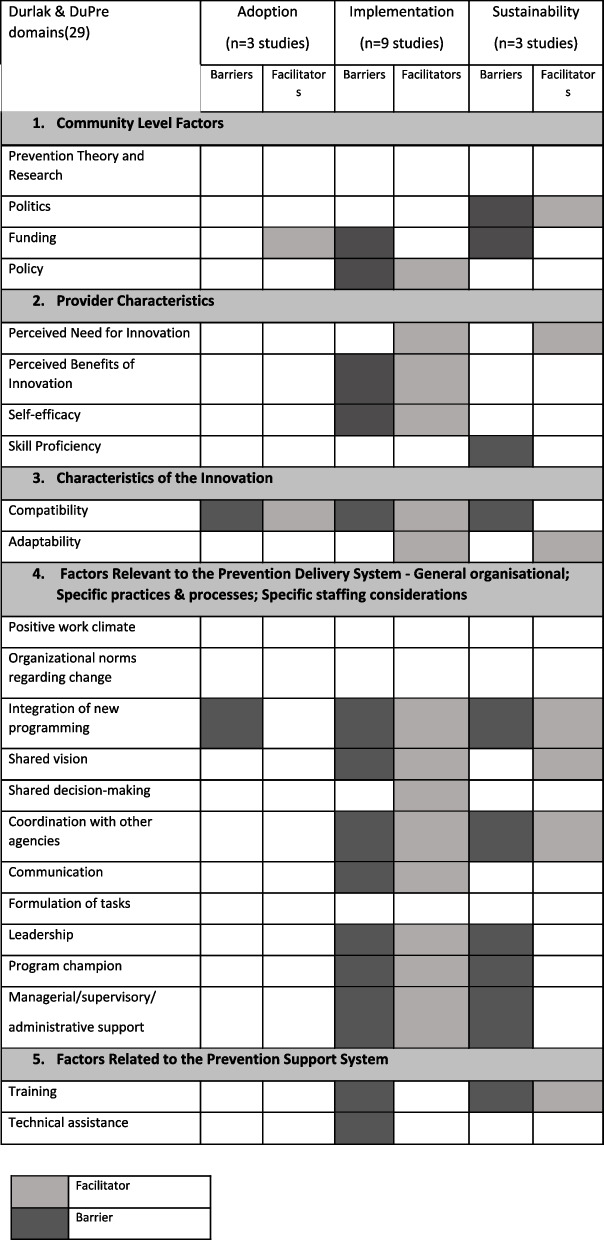
Barriers and facilitators influencing intervention adoption, implementation and sus-tainability categorised by Durlak and DuPre [29]

#### Intervention adoption

Within the intervention adoption phase, three factors: ‘funding’, ‘compatibility’ and ‘integration into new programming’ were identified as barriers/facilitators across three Durlak and DuPre domains. ‘Funding’ and ‘compatibility’ were most identified as facilitators affecting adoption. Compatibility’ was also identified as a barrier as well as ‘integration of new programming’.

#### Intervention implementation

Seventeen factors were identified within the intervention implementation phase across all five Durlak and DuPre domains. ‘Perceived benefits of the innovation’ and ‘integration of new programming’ were the most identified facilitators for intervention implementation. ‘Compatibility’ was the most identified barrier (e.g., competing activities, scheduling difficulties), followed by ‘managerial/supervisory/administrative support’ (e.g., high staff turnover and managers expectations). Eleven of the seventeen identified factors were described as both a barrier and a facilitator, most notably self-efficacy, compatibility, and Managerial/supervisory/administrative support.

#### Intervention sustainability

Thirteen factors were identified within the intervention evaluation phase across all five Durlak and DuPre domains. ‘Politics’ (e.g., government leadership and political support) and ‘adaptability’ (e.g., flexibility and adaptations to training, interpersonal communication, and resources) were most identified as facilitators for intervention sustainability. ‘Politics’ (e.g., unreliable public sector fund allocation, increasing privatization of the health sector) was also described as a primary barrier, as well as ‘funding’ (e.g., concerns about the requisite financial resources to sustain program activities). Four of the thirteen identified factors were described as both a barrier and a facilitator, namely, ‘politics’, ‘integration into new programming’, ‘coordination with other agencies’, and ‘training’.

## Discussion

This paper reviewed the use of implementation science theories, models or frameworks (TMFs) reported by early childhood feeding, nutrition and active play interventions implemented under real-world settings. It also identified key barriers and facilitators affecting adoption, implementation and sustainability of these interventions. Eleven studies were identified for inclusion, representing four of the five Nilsen [21] implementation model categories, with evaluation frameworks and implementation theories used most frequently. RE-AIM was the only framework used multiple times. Studies mainly reported on barriers and facilitators to the intervention implementation and sustainability phases, with few studies reporting on the intervention adoption phase.

### The application of TMFs in early childhood (< 5 years) feeding, nutrition and active play interventions

The importance of the application of TMFs during the planning, implementation and/or evaluation of interventions is well documented in the literature [21, 36, 37, 40, 44, 47, 48]. The alignment of identified TMFs of included studies with four of Nilsen’s five implementation model categories is promising as the success of intervention implementation and scale up is enhanced by the application of concepts from implementation science [21, 49]. The challenge remains however, to enhance uptake and application of TMFs within early childhood intervention research to enhance real-world implementation success. The apparent limited uptake of TMFs is a lost opportunity for a more complete understanding of intervention and implementation outcomes.

The identification of RE-AIM as the most cited TMF aligns with findings from previous reviews. In a narrative review of frameworks used for translating evidence into policy and practice, 17 of the 41 included studies used the RE-AIM framework [50]. Similarly, in a systematic review of school-based physical activity and sedentary behaviour interventions, Cassar [51] reported three of 14 included studies used the RE-AIM framework. A review specific to TMFs within childcare settings identified RE-AIM was used in evaluation of two of the 38 included studies [52]. Traditionally, RE-AIM is an implementation evaluation framework used to identify and evaluate aspects of the implementation process [53] and as such, provides essential guidance to the success of implementation strategies and potentially intervention outcomes. Three of the four studies in our review, that used RE-AIM, did not explicitly pair its use with a theoretical basis, or a determinant or process framework, leaving scope for enhanced application of implementation science principles more broadly across the lifespan of these studies [36, 37, 44].

A review of implementation science TMFs in 2018 identified 159 TMFs that had been used to guide dissemination or implementation of evidence-based interventions of cancer or chronic diseases [54]. Of concern, 60% of these were only used once. Despite this broad range of existing models, there remains a real or perceived need to create new TMFs for implementation evaluation [34, 39], or to adapt existing frameworks, for example, the Yamey framework [46] adapted from the Theory of Diffusion [21, 46, 55]. It is likely that the creation of new TMFs arises due to the plethora of existing TMFs and the related difficulty of choosing the most relevant TMF [56].

Our findings are in line with current literature demonstrating that there is no systematic application in the planning, implementation and/or evaluation of interventions in real-world settings and that there is a selective and sporadic application of TMFs across the lifespan of interventions [51, 54]. Furthermore, the literature demonstrates it is uncommon for interventions to use a TMF, with a majority of scaled up intervention trials not applying a TMF [47, 57, 58]. This makes the process of translating evidence-based trials into practice challenging and misses an important opportunity to follow existing structured guidance to facilitate replication.

### Barriers and facilitators pertinent to the adoption, implementation and sustainability of early childhood (< 5 years) feeding, nutrition and active play interventions

It is recognised that successful outcomes relating to the adoption, implementation and sustainability of an intervention within a real-world setting is dependent on identifying barriers and facilitators [21, 50]. The Durlak and DuPre framework [30] identified facilitators and barriers across all three intervention phases of adoption, implementation and sustainability, with factors relating to the implementation phase most frequently reported. This finding aligns with others who suggest limited literature is available on intervention adoption [59] and intervention sustainability [60]. The translation of research into practice begins with adoption and succeeds with sustainability, therefore a lack of research regarding factors affecting these intervention phases limits the development of tailored, phase-specific implementation strategies.

‘Compatibility’, ‘integration of new programming’ and ‘funding’ were commonly cited as both facilitators and barriers across all three intervention phases. As reflected within the literature [51, 61, 62], multiple factors are reported as both a facilitator and a barrier in relation to intervention adoption, implementation and sustainability. The common citing of ‘compatibility’ and ‘adaptability’ as facilitators of intervention implementation highlights the value placed on contextualisation and consideration of diversity when developing and sustaining interventions [25, 26, 63, 64]. ‘Funding’, cited as a barrier and a facilitator across the intervention lifespan, illustrates how this resource is considered essential for the initiation and continued implementation of interventions [31, 62, 63, 65, 66].

### Implications for research and practice

There is little guidance providing a practical and systematic approach to effectively planning intervention implementation and scale up specific to early childhood feeding, nutrition and active play interventions in real-world settings. This poses challenges for the transfer of research into practice and may indicate a gap in the literature [21, 48, 65].

To minimise this challenge, it is suggested that implementation science TMFs be used in combination across the lifespan of the intervention to create rigor to the planning, implementation and sustainability in real world settings and contexts [21, 48, 65, 67, 68].

It is also apparent that the identification of barriers and facilitators across the intervention phases is important to enhance the likelihood of successful adoption, implementation and sustainability in a real-world setting. It is therefore imperative for researchers and practitioners to consider and include appropriate measures across the lifespan of an intervention [21, 50], particularly in relation to funding and compatibility, which have the potential to change impact or need, based on the intervention setting. Gaining deeper understanding of these factors, particularly those that are underreported such as shifting organisational norms and positive work climate, warrants further research.

### Strengths and limitations

A major strength of this systematic review includes the presence of studies with interventions in low-middle- and high-income countries allowing the findings to be relevant across a range of settings and demographics. This review also utilised reputable implementation science TMFs for data extraction and analysis, namely Nilsen [21] and Durlak and DuPre [30].

Study selection and data extraction was conducted using Covidence software which facilitated cross-checking by multiple authors and maintained quality control. The Mixed Methods Appraisal Tool (MMAT) enabled all included studies to be appraised using a single tool that accommodated all study designs.

It is apparent from this review, that studies applying TMFs for early childhood health behaviour interventions in real-world settings are scarce, and as such, it is difficult to generalise our findings across multiple settings, communities, and populations. This review helps to identify the barriers and facilitators of implementation in the real-world setting to encourage adaptation and highlight what may or may not affect practice. This review reiterates the difficulties experienced in translating efficacy trials to real-world settings and identifies the barriers and facilitators of implementation.

This review included studies with demonstrated positive outcome measures on diet, physical activity and sedentary behaviour. As a result, efficacy trials were excluded. This may have resulted in exclusion of implementation trials, although one hybrid effectiveness-implementation trial was identified in the search and included. The usage of TMFs, and barriers and enablers to implementation, within implementation trials exploring implementation strategies may therefore have been missed.

## Conclusion

Various challenges accompany the transfer of research from a controlled (trial) environment into a real-world setting, indicating an evidence gap and suggesting that a theory driven approach throughout the lifespan of an intervention could enhance its design, adoption, implementation and scale-up. Findings demonstrate that there is no systematic application of TMFs in the planning, implementation and/or evaluation of early childhood nutrition and active play interventions in real-world settings, and selective and sporadic application of TMFs occurs across the intervention lifespan. This apparent limited uptake of TMFs is a missed opportunity to enhance real-world implementation success.

It is recommended that research exploring the adoption, implementation and sustainability of early childhood health behaviour interventions be conducted using TMFs more systematically to enhance the adoption, implementation and sustainability of early childhood feeding, nutrition and active play interventions in real-world settings.

## Supplementary Information

**Additional file 1. **

**Additional file 2. **

**Additional file 3. **

**Additional file 4. **

## Data Availability

Not applicable.

## Abbreviations

TMFs: Theories, models and frameworks
PRISMA: Preferred Reporting Items for Systematic Reviews and Meta-Analyses
MMAT: Mixed Methods Appraisal Tool
WIC: Women, Infants, and Children
PIP: Program Impact Pathway
MRC: Medical Research Council
i-PARIHS: The integrated Promoting Action on Research Implementation in Health Services
RE-AIM: Reach, Effectiveness, Adoption, Implementation, Maintenance
