# Supplementary material for: Adoption, implementation, and sustainability of early childhood feeding, nutrition and active play interventions in real-world settings: a systematic review
Source: Int J Behav Nutr Phys Act. 2023 Mar 20;20:32. doi: 10.1186/s12966-023-01433-1 (PMC10029282; doi:10.1186/s12966-023-01433-1)
Supplement: Supplementary file 1 — Additional file 1. [file 12966_2023_1433_MOESM1_ESM.docx]

**Appendix 1: Search Strategy Example**

EMBASE

| **No.** | **Query** | **Results** |
| --- | --- | --- |
| #62 | #61 AND (2000:py OR 2001:py OR 2002:py OR 2003:py OR 2004:py OR 2005:py OR 2006:py OR 2007:py OR 2008:py OR 2009:py OR 2010:py OR 2011:py OR 2012:py OR 2013:py OR 2014:py OR 2015:py OR 2016:py OR 2017:py OR 2018:py OR 2019:py OR 2020:py) | 1450 |
| #61 | #4 AND #33 AND #56 AND #58 AND #59 AND #60 | 1428 |
| #60 | #34 OR #35 OR #36 OR #37 OR #38 OR #39 OR #40 OR #41 OR #42 OR #43 OR #44 OR #45 OR #46 | 540369 |
| #59 | #17 OR #18 OR #19 | 2359081 |
| #58 | #5 OR #6 OR #7 OR #8 OR #9 OR #10 OR #11 OR #12 OR #13 OR #14 OR #15 OR #16 OR #57 | 3623994 |
| #57 | 'body mass index':ab,ti | 257525 |
| #56 | #47 OR #48 OR #49 OR #50 OR #51 OR #52 OR #53 OR #54 OR #55 | 8451565 |
| #55 | concept*:ab,ti | 569404 |
| #54 | manual*:ab,ti | 167639 |
| #53 | guideline*:ab,ti | 529316 |
| #52 | protocol*:ab,ti | 628218 |
| #51 | strateg*:ab,ti | 1350643 |
| #50 | approach*:ab,ti | 2158973 |
| #49 | plan*:ab,ti | 1431554 |
| #48 | model*:ab,ti | 3543673 |
| #47 | framework*:ab,ti | 289763 |
| #46 | 'service delivery':ab,ti | 16220 |
| #45 | 'routine practice*':ab,ti | 11463 |
| #44 | 'research to practice':ab,ti | 2736 |
| #43 | 'real-world':ab,ti | 65658 |
| #42 | 'rolled out':ab,ti | 1433 |
| #41 | 'roll out':ab,ti | 2362 |
| #40 | translat*:ab,ti | 378975 |
| #39 | 'scale out':ab,ti | 190 |
| #38 | scalability:ab,ti | 5501 |
| #37 | scaling:ab,ti | 46342 |
| #36 | 'scaling up':ab,ti | 5686 |
| #35 | 'scaled up':ab,ti | 5236 |
| #34 | 'scale up':ab,ti | 14193 |
| #33 | #20 OR #21 OR #22 OR #23 OR #24 OR #25 OR #26 OR #27 OR #28 OR #29 OR #30 OR #31 OR #32 | 8697601 |
| #32 | evaluat*:ab,ti | 4799523 |
| #31 | feasib*:ab,ti | 441819 |
| #30 | sustain*:ab,ti | 444431 |
| #29 | utilization:ab,ti | 244830 |
| #28 | utilisation:ab,ti | 24639 |
| #27 | modif*:ab,ti | 1288730 |
| #26 | adapt*:ab,ti | 635951 |
| #25 | adopt*:ab,ti | 301279 |
| #24 | uptake:ab,ti | 444742 |
| #23 | delivery:ab,ti | 578415 |
| #22 | diffus*:ab,ti | 482292 |
| #21 | disseminat*:ab,ti | 161692 |
| #20 | implement*:ab,ti | 621839 |
| #19 | initiative*:ab,ti | 117847 |
| #18 | intervention*:ab,ti | 1334347 |
| #17 | program*:ab,ti | 1127209 |
| #16 | 'energy balance':ab,ti | 17216 |
| #15 | 'healthy lifestyle*':ab,ti | 10503 |
| #14 | 'obesity prevention':ab,ti | 4710 |
| #13 | bmi:ab,ti | 299582 |
| #12 | play:ab,ti | 830872 |
| #11 | active*:ab,ti | 1250617 |
| #10 | 'sedentary time':ab,ti | 2795 |
| #9 | 'physical activity':ab,ti | 141111 |
| #8 | nutrition:ab,ti | 210518 |
| #7 | diet*:ab,ti | 707860 |
| #6 | 'feeding practice*':ab,ti | 5758 |
| #5 | feed*:ab,ti | 507952 |
| #4 | #1 OR #2 OR #3 | 2139844 |
| #3 | child*:ab,ti | 1784572 |
| #2 | 'early childhood':ab,ti | 32304 |
| #1 | infan*:ab,ti | 532042 |
