# Supplementary material for: Adoption, implementation, and sustainability of early childhood feeding, nutrition and active play interventions in real-world settings: a systematic review
Source: Int J Behav Nutr Phys Act. 2023 Mar 20;20:32. doi: 10.1186/s12966-023-01433-1 (PMC10029282; doi:10.1186/s12966-023-01433-1)
Supplement: Supplementary file 4 — Additional file 4. [file 12966_2023_1433_MOESM4_ESM.docx]

| **ADOPTION** | |
| --- | --- |
| **Facilitators** | **Barriers** |
| **1. Community Level Factors**  **Funding**   - Running free session identified as a major incentive for participation (4) | **1. Community Level Factors** |
| **2. Provider Characteristics** | **2. Provider Characteristics** |
| **3. Characteristics of the Innovation**  **Compatibility**   - Participant commitment due to large time investments (3) - Face-to-face training is highly valued in remote Aboriginal and Torres Strait Islander settings by both participants and facilitators. (6) | **3. Characteristics of the Innovation**  **Compatibility**   - Lack of clarity with content and design of the sessions (3) |
| **4. Factors Relevant to the Prevention Delivery System: Organisational Capacity** | **4. Factors Relevant to the Prevention Delivery System: Organisational Capacity**    **General organisational factors:**  **Integration of new programming**   - Lack of building infrastructure and lack of space (4) |
| **5. Factors Related to Prevention Support System** | **5. Factors Related to Prevention Support System** |
| **Total No. 3 factors** | **Total No. 2 factors** |

| **IMPLEMENTATION** | |
| --- | --- |
| **Facilitators** | **Barriers** |
| **1. Community Level Factors**  **Prevention theory and research**   - evidence base underpinning the program gave them the confidence to have supportive conversations (8)   **Funding**   - Financial incentives (11)   **Policy**   - Policies (6) | **1. Community Level Factors**  **Funding**   - Lack of salary and incentives (2) - lack of incentives (9) - Lack of funding (2)   **Policy**   - center policies negatively influencing implementation (5) |
| **2. Provider Characteristics**  **Perceived need for innovation**   - Met social need: social recognition (9)   **Perceived benefits of innovation**   - Perceived improvements in staff communication and teamwork. (1) - Perceptions of changes in clinics resources, practices, and attitudes related to breastfeeding promotion (1) - Improvements with mother/ child interaction (2) - Sense of community/ peer support (2) - Improvements to children's nutrition (2)   **Self-Efficacy**   - Self-identiﬁed areas for improvement and gaps in knowledge (1) (8) - implementation was dependent on having the right staff, training them in how to facilitate the program, and allowing them to do their jobs (4)   **Skill proficiency**   - opportunity to acquire knowledge and skills (9) | **2. Provider Characteristics**  **Perceived benefits of innovation**   - lack of time (5) - lack of parent engagement (5) - Lack of incentives (10) (11)   **Self-efficacy**   - Self-reported high level of knowledge at baseline, so there was limited opportunity for improvement (8) - Lack of guidelines and expertise (9)   **Skill proficiency**   - lack of skills in promoting the initiative (11) |
| **3. Characteristics of the Innovation**  **Compatibility**   - Targeted group discussions were reported as being well received by both staff members and attending participants. (1)   **Adaptability**   - Flexibility to conduct both one-to-one and group sessions (2) - Adaptations to the timing of program and to program resources (4) (8) - Strong organization skills and flexibility in working arrangements (9) | **3. Characteristics of the Innovation**  **Compatibility**   - Low attendance (1) - Lack of group interaction (4) - lack of time (4) - Lack of need (4) - competing activities, scheduling difficulties, family problems, poverty, transportation issues (4) - Lack of participant motivation to attend weekly sessions. (4) - Lack of financial and material resources (9) |
| **4. Factors Relevant to the Prevention Delivery System: Organisational Capacity**    **General organisational factors:**  **Integration of new programming**   - clinic-wide implementation policies (1) - activities integrated with existing requirements (5) - The embeddedness of the service within the community (6) - integrated into the existing health system (9) - mutual reinforcement between the grain bank activities and the health sector activities (9)   **Shared vision**   - Communities expressed a strong sense of ownership towards the project (9)     **Specific practices and processes:**  **Shared decision making**   - Getting staff buy-in. (1)   **Coordination with other agencies**   - Explanation of the project to community members and leaders. (2)   **Communication**   - Provided opportunities for building capacity of Early Childhood Education and Care (ECEC) staff to act as community resources (6) - Effective communication, learning, and mentoring between ECEC staff. (6) - Effective communication (11)     **Specific staffing considerations:**  **Leadership**   - trusting relationship between the lead facilitator had developed with the CHN service (8)   **Program champion**   - Parent’s volunteering (2) - Champions (9) (11)     **Managerial/supervisory/administrative support**   - creating implementation protocols to support and enable both staff and participants to adhere to the scheduled appointments. (1) - Strategies aimed at participants included reminder pamphlets, letters, text messages, and phone calls to mothers. (1) - Having managers onboard who are also members of the research team so can provide support for the research by encouraging attendance and engagement with the program (8) | **4. Factors Relevant to the Prevention Delivery System: Organisational Capacity**    **General organisational factors:**  **Integration of new programming**   - time/commitment (2) - Competing priorities (2) - a crossover between professional and personal relationships within small communities (6)   **Shared vision**   - Formal Weaknesses Commitment Form And so, it was still being explained to me you know…what it was and making sure that I understood the form before I even signed it.” (11)     **Specific practices and processes:**  **Coordination with other agencies**   - Coordination gap between agencies (9) (10)   **Communication**   - Lack of awareness and motivation (10)   **Specific staffing considerations:**  **Leadership**   - Competing priorities from healthcare workers (e.g., vaccine, child health week, or HIV campaigns) (2) - incentives by research studies to do other work or get training (2)   **Program champion**   - increasing workload affected supervision and monitoring of the grain bank and occasionally caused interruption of complementary food production and distribution. (9)     **Managerial/supervisory/administrative support**   - Some key service staff are often undertaking multiple roles within the community making (6) - Poor planning (9) - High Staff turnover (9) - Manager’s expectations of facilitators (11) |
| **5. Factors Related to Prevention Support System** | **5. Factors Related to Prevention Support System**  **Training**   - Delayed training caused loss of momentum (9) - lack of refresher training for staff (9)   **Technical assistance**   - logistical barriers eg. slow internet speeds (4) |
| **Total No. 33 factors** | **Total No. 33 factors** |

| **SUSTAINABILITY** | |
| --- | --- |
| **Facilitators** | **Barriers** |
| **1. Community Level Factors**  **Prevention theory and research**  **Politics**   - Government leadership and involvement at all levels (7) - Political support (9) | **1. Community Level Factors**  **Prevention theory and research**  **Politics**   - Unreliable public sector fund allocation (7) - Increasing privatization of the health sector (7) - omitting Infant and Young Child Feeding (IYCF) counseling from the national health insurance program. (7)   **Funding**   - Lack of funding or partnerships (4) - limited or a lack of incentives (7) - concerns about the requisite financial resources for sustaining program activities (7) |
| **2. Provider Characteristics**  **Perceived need for innovation**   - High perceived need (4) | **2. Provider Characteristics**  **Perceived need for innovation**   - waning interest from caregivers (7)   **Skill proficiency**   - high knowledge scores did not translate into better counseling (7) |
| **3. Characteristics of the Innovation**  **Adaptability**   - adaptations to training, interpersonal communication and resources (7) - flexibility (7) | **3. Characteristics of the Innovation**  **Compatibility**   - Lack of equal access (9) |
| **4. Factors Relevant to the Prevention Delivery System: Organisational Capacity**    **General organisational factors:**  **Integration of new programming**   - Integration into the existing health system (9)   **Shared vision**   - Partnership, including with civil society, and wide stakeholder buy-in (7)     **Specific practices and processes:**  **Coordination with other agencies**   - The multi-pronged design utilizing different organisations (7)     **Specific staffing considerations:** | **4. Factors Relevant to the Prevention Delivery System: Organisational Capacity**    **General organisational factors:**  **Integration of new programming**   - competing priorities (7) - Time constraints (7)   **Shared vision**  **Specific practices and processes:**  **Coordination with other agencies**   - varied activities require expertise and support from different implementing agencies (7) - coordination challenges (7)   **Specific staffing considerations:**  **Leadership**   - low IYCF knowledge among new leadership (7) - Requires leadership from different implementing agencies (7)   **Program champion**   - concerns about the continuity and long-term commitment of champions (7) - loss of institutional knowledge due to high turnover (7)   **Managerial/supervisory/administrative support**   - Turnover of human resources, at both leadership and implementation levels (7) |
| **5. Factors Related to Prevention Support System**  **Training**   - Vigorous training (7) | **5. Factors Related to Prevention Support System**  **Training**   - training efforts have decreased in terms of frequency, type and mechanisms for ongoing education and support. (7) |
| **Total No. 9 factors** | **Total No. 19 factors** |

Eldridge (1), Gladstone (2), Harms (3), Heerman (4), Luecking (5), Murtha (6), Moucherard (7), Norton (8), Sako (9), Sarma (10), Swindle (11)
